# Mr. Park on the Progress of Vaccination in Liverpool

**Published:** 1811-04

**Authors:** 


*To the Editors of the Medical and Physical Journal.*

GENTLEMEN,

THE following letter, which I lately received from Mr. Park, an eminent surgeon of Liverpool, shews the progress of vaccination in that part of the kingdom.

"DEAR SIR,

"Please to accept my best thanks for your politeness, in sending me your publications on the subject of vaccination; with my warmest wishes for the success of your exertions, in defence of that practice, against the attacks of its enemies; and I shall be truly happy to be assured by you, that there is no foundation for the reports which I hear circulated in this place, that the practice in the metropolis is on the decline. Of the superior advantages of this to the variolous inoculation I conceive no one can entertain a doubt, who will view the subject with an unprejudiced eye; and derive his ideas from that source, which could have no other object in view than the full investigation of truth. In this light only can we consider the inquiry of parliament into the practice; their proceedings clearly prove their conviction of its superiority, although they admit of some failures. If I remember right (for I have not their publication at present in my possession) they admit the possibility of one in 150 being left susceptible of the infection of the small pox; whereas it is allowed that nearly that proportion die under inoculation with variolous matter. But, in drawing a comparison between the advantages arising to the community from one or the other inoculation, there is a more important question which I have not yet seen fairly agitated; viz. if vaccination had never been discovered, is it certain, or even probable, bable, that variolous inoculation would have been practised to the same extent? Judging from what has passed in Liverpool, I can say decidedly, No:—but that I may not be thought to speak at random, I will state the data on which I ground this declaration. About twenty-eight years ago somewhat more than twenty of us, who were doing the principal business in Liverpool, formed ourselves into a society for the gratuitous inoculation of the poor at their own houses. We divided the town into districts, each taking one; and canvassed every house from garret to cellar, to offer and recommend inoculation. The annual births were then about 1800; and we found somewhat more than 4000 persons who had not had the disease. Of these we inoculated upwards of 400 the first year; the second not much above 300; and each succeeding year below 300, including private as well as gratuitous patients. Upon this we dissolved the society; giving one final general notice, that we should always be ready in the months of March and October, to inoculate gratis all such as should offer or be recommended as proper objects. From that time till the introduction of vaccination, gratuitous inoculation fell almost into disuse. Scarcely half a dozen applied to me in a year for the purpose; and I am confident the numbers never equalled one sixth of the births, under the increasing population, if they even equalled the gross number, at the time of the dissolution of our society. But as soon as vaccination became popular, the numbers increased very much indeed. In the beginning of the year 1807, I collected the best account I could, for the information of the College of Surgeons; although I could not get any statements from several of the practitioners, nor can I pledge myself for the perfect correctness of all that I did obtain; but it appeared that the gross numbers for the year 1806 were more than 2,300, exclusively of some hundred American seamen. My own number that year was only 402. Since that it has increased as follows; for 1807, 1010; for 1808, 1465; for 1809, 1011; and for the current year to the time of writing this, Nov. 8th, 1530. The births are now between 3,700, and 3,800. From this it will clearly appear, that if the other practitioners, and the public charities, continue to vaccinate in the proportion which they did in 1806, the gross numbers must now exceed 3000 annually; or be equal to five-sixths of the births, under the increased population. This will clearly enable any unprejudiced man to form a tolerable estimate, or at least a very probable conjecture, of the great superiority of numbers that must be left exposed to the ravages of the natural small-pox, if vaccination had not been introduced. It may not be amiss amiss here to state, that of the first 1300 inoculated by our society with variolous matter, seven died; and although I have not been so unfortunate as to have one of those deaths fall to my lot, yet I have suffered much greater alarms for the safety of my patients, and experienced much more distressing sequels from that inoculation, than I ever did from vaccination; although I have vaccinated considerably more than double the number of patients during the four last years, that I ever inoculated with variolous matter during the preceding forty years. Now to return to the admission in the parliamentary reports, that one in one hundred and fifty may have been left susceptible of the natural small-pox, I think my own experience will justify me in expressing a confident hope, that this must have been founded on some erroneous statements presented to them; although it is only to the result of my private vaccinations that I can speak with any degree of certainty. These have never amounted to more than between sixty and seventy in a year, out of the gross numbers which I have stated above; and the total amount of them falls somewhat short of seven hundred. Of these, however, I can confidently say, not a single instance has yet come to my knowledge, of the small-pox occurring after vaccination; although, for two or three of the first years, I inoculated nearly all my patients over again with variolous matter, but without being able to produce disease. Of my gratuitous patients, which amount to upwards of 5000, I cannot speak with the same certainty. They are wholly unknown to me; are vaccinated in my own house; and a considerable proportion of them never present themselves again for inspection. I can, however, say with truth, that only three have yet come to my knowledge, whose mothers say I expressed myself satisfied with their vaccine vesicles, that have since had a disease which might be taken for small-pox; but whether it really was so or not I submit to your judgment, when I give you as faithful a description of them as I can; only saying that they were such a kind of pock as I would not use for inoculation; but have repeatedly rejected when variolous inoculation only was in use. The crops were nearly as copious as could exist, to remain perfectly distinct. They had all the uniformity and purulence of small-pox; but were not so fleshy about the base; being more vesicular, and, though so copious, the greater part of them turned in the face by the end of the sixth day, and in turning did not encrust, but bursted and fell flat on the skin; whereas I had an opportunity, at the same time, of comparing one of them with a crop similar in number, in a young woman, a nurse in a family, who had never been inoculated.

inoculated. Of these very few in the face turned till the eleventh day; and in turning formed a crust nearly half the size of the pustules in their mature state. I cannot conclude this letter without saying I have been much disappointed with the proceedings of parliament on this occasion. When they had the subject twice before them, I did expect they would have passed some act restraining variolous inoculation, at least to stated periods, and circumstances of certain seclusion from the mass of society; and not have left medical men, who are straining every nerve for the preservation of the lives of their fellow creatures, completely in the lurch, to contend the point by themselves, unsupported by any act of the legislature. Should parliament, at any future time, think it adviseable to pass any act of this nature, I see no reason why it may not extend to a similar seclusion of persons affected with the natural small pox; or even with typhus, scarlatina, and other destructive epidemics.

I am, Sir, with due regard,

Your obedient Servant,

H. PARK."

*Liverpool, Nov.* 9, 1810.

---

Mr. Park is mistaken in supposing parliament to have admitted, that one person in one hundred and fifty may be left susceptible of the small-pox. Parliament did not enter into any calculation of that kind. Mr. Rose expressed an opinion, that in consequence of the distribution of bad matter, about one failure happened in three hundred cases. This opinion, however, appears to have been erroneous; and another was equally so, that the Jennerian Society was so poor as not to be able to distribute matter equal to the demand.

The Ringwood cases, which Mr. Rose mentioned in proof of his argument, did not warrant such a conclusion; since it appears, by the report signed by Mr. Rose himself, as well as by the professional men who assisted him in his investigation, that not a single failure occurred there.

It is rather remarkable, that Mr. Park has not happened to see the question, relative to the prodigious extension of inoculation, in consequence of Dr. Jenner's discovery, fairly agitated. That question forms a distinguished feature in the works of all the principal writers in favor of vaccination, and in those of their opponents; the former rejoicing, and the latter repining at this event. It is also particularly noticed in the Report of the College of Physicians; where it is observed, that the practice of vaccination had spread with unexampled rapidity.

By a subsequent letter, Mr. Park informs me, that the total amount of his vaccinations for last year was 1720. He also states, that he has now met with one case, which he is convinced was a failure. The small-pox, though rather plentiful, was, as usual, so far mitigated, that it turned about the sixth day. He declares, however, that he has repeatedly seen a recurrency of the same sort of small-pox after variolous inoculation; and that he can produce as many cases of the small-pox occurring a second time, as have occurred, at Liverpool, after vaccination.

Having often experienced a great inconvenience from the want of a constant supply of matter, in consequence of so many of the patients neglecting to attend, Mr. Park has at length resolved to discontinue gratuitous vaccination at his own house; but he has provided a successor, Mr. Dawson, who is also a respectable surgeon, and a member of the College. Mr. Dawson has undertaken to vaccinate at his own house; and as he is surgeon to the Dispensary, and to the Lying-in-Charity, where vaccination is particularly enjoined, his influence will be the greater.

I am, Gentlemen,

Yours, &c.

*New Street, Hanover Square,*
*March* 4, 1811.

